# Continuous versus Intermittent Enteral Feeding in Critically Ill Children: A Systematic Review

**DOI:** 10.3390/nu15020288

**Published:** 2023-01-06

**Authors:** Xenophon Theodoridis, Lydia Chrysoula, Kleo Evripidou, Ioustini Kalaitzopoulou, Michail Chourdakis

**Affiliations:** Laboratory of Hygiene, Social and Preventive Medicine and Medical Statistics, School of Medicine, Faculty of Health Sciences, Aristotle University of Thessaloniki, 54124 Thessaloniki, Greece

**Keywords:** enteral nutrition, bolus, continuous, pediatric, systematic review

## Abstract

Administration of enteral nutrition (EN) in critically ill pediatric patients admitted to the pediatric intensive care unit (PICU) constitutes a major challenge due to the increased risk of complications, as well as the lack of well-trained healthcare professionals. EN is usually delivered via cyclic, continuous, or intermittent feeding; however, a number of potential barriers have been reported in the literature regarding different feeding regimens. The purpose of this review was to assess the effectiveness of continuous and intermittent bolus feeding on critically ill children. A systematic search was conducted in PubMed, Scopus Cochrane Central Register of Controlled Trials (CENTRAL) and a clinical trial registry up to September 2022, including randomized controlled trials (RCTs) published in the English language. Four studies met the inclusion criteria with a total population of 288 patients admitted to the PICU. Three studies were rated with a high risk of bias and one with some concerns. There was high heterogeneity between the studies in regard to the reporting of outcomes. Three studies measured the total time needed to reach prescribed caloric intake with conflicting results, while two studies evaluated the length of stay (LOS) in PICU with no difference between the two arms. One study assessed the time weaning from mechanical ventilation, favoring the bolus group. No data were provided for gastric residual volume (GRV), anthropometric measurements, and biochemical markers. Additional randomized trials with better methodology are needed to assess the efficacy of the two enteral feeding regimens in critically ill PICU patients.

## 1. Introduction

Children admitted to the pediatric intensive care unit (PICU) are at increased risk for malnutrition [1]. A deteriorated nutritional status has been associated with a plethora of negative clinical outcomes, including a higher risk for extended need for mechanical ventilation, muscle weakness, impaired immune response, increased susceptibility to infections, as well as higher morbidity and mortality [2]. Prolonged PICU stay and hospitalization, as well as increased healthcare costs, underline the detrimental impact of malnutrition on patients’ health status and the public health systems, respectively [3,4]. Moreover, an excessive caloric delivery may be baneful in the acute phase of critical illness due to inefficient substrate use [5].

Feeding a critically ill child is a complex and challenging procedure and, in certain scenarios, may be characterized as a fruitless effort [6]. Enteral nutrition (EN) is considered the preferred method of feeding in patients with a functioning gastrointestinal (GI) tract due to its multiple benefits regarding physiological aspects, including maintenance of the functional integrity of the GI tract, improved injury healing, decreased catabolic responses, reduced inflammation, as well as decreased risk of bacterial colonization and infections [7]. However, several studies indicate that the provision of enteral nutrition (EN) can be suboptimal, with the main difficulties in achieving the caloric target being intolerance to artificial feeding and increased risk of aspiration pneumonia [8]. Nutrition therapy interruptions constitute an additional challenge during the provision of artificial nutrition, which can occur due to reintubation, continuous episodes of vomiting and diarrhea, hemodynamic instability, displacement of feeding tubes, tube blockage, or due to medical examination of the patient [9]. In addition, the lack of an established nutrition support team and well-trained healthcare professionals to feed patients, limited personnel, especially on weekends or evenings, and decreased adherence to clinical practice guidelines are among the barriers that have been identified with regard to the provision of effective and optimal delivery of nutrition care in ICU [10,11].

Artificial nutrition can be administered via continuous, cyclic, intermittent, or bolus feedings. Advantages of continuous feeding include a potential tolerance improvement and reduced risk of aspiration. Contrariwise, it is a costly technique requiring a feeding pump and having several practical limitations during rehabilitation and other related procedures [12]. On the other hand, the intermittent or bolus methods are more physiologic and do not depend on a feeding pump. However, they increase the risk of aspiration, lead to glucose variability, and may delay gastric emptying time leading in some cases to increased gastric residual volume (GRV) [12]. Furthermore, intermittent EN administration, in comparison to continuous feeding, seems to improve protein synthesis in critically ill adults [13].

Given the documented barriers to feeding and the paucity of evidence with regard to the efficacy of the different methods of EN administration in PICU patients, it remains unclear which is the most appropriate feeding regimen for reaching nutritional target goals and improving patients’ nutritional status, minimizing potential adverse effects, length of mechanical ventilation, and hospital stay. Therefore, we performed a systematic review to evaluate the effectiveness of continuous and intermittent EN feeding on nutritional and PICU outcomes in critically ill children. 

## 2. Materials and Methods

This systematic review was performed according to the Preferred Reporting Items for Systematic Reviews and Meta-Analyses (PRISMA) checklist [14] (Supplementary Materials, Table S1) and the synthesis without meta-analysis (SWiM) guidelines [15]. A detailed protocol has been registered in PROSPERO with ID CRD42022359184.

### 2.1. PICO and Search Strategy

We performed a systematic search from inception until September 2022 in electronic databases such as PubMed, Scopus, Cochrane Central Register of Controlled Trials (CENTRAL), a clinical trial registry (clinicaltrials.gov, last accessed on 26 December 2022), and gray literature. Reference lists from previous systematic reviews and meta-analyses were also screened manually for the identification of eligible studies. Only original studies published in the English language without a restriction on publication date were included. A basic search string was developed for PubMed and modified accordingly for all databases. The search terms used for identifying the relative literature were “enteral feeding”, “enteral nutrition”, “tube feeding”, “artificial nutrition”, “child”, “pediatric”, “infant”, “adolescent”, “youth”, “randomized”, and “randomized controlled trial”. The full syntax of the search string can be found in Table S2.

### 2.2. Inclusion Criteria

For this review, we included studies that met the following criteria: (1) randomized controlled trials (RCTs), (2) including critically ill pediatric population (<18 years old), (3) comparing the administration of bolus-enteral nutrition or cyclic-enteral nutrition, or continuous enteral nutrition, and (4) published in the English language.

### 2.3. Exclusion Criteria

Studies with a different study design, including non-randomized clinical trials, were performed on pediatric patients who received only parenteral nutrition (peripheral or total) or supplemental parenteral nutrition. Patients diagnosed with major congenital malformation, short bowel syndrome (SBS), malabsorption syndrome, and metabolic disease requiring a specific diet were excluded. Studies including infants with low birth weight (LBW) and very low birth weight (VLBW) were also excluded from this review. We also excluded studies lacking an active comparator group, such as bolus-enteral nutrition, cyclic, or continuous enteral nutrition. No restrictions were imposed on intervention duration.

### 2.4. Data Extraction

Data extraction was performed independently by three reviewers using a structured, piloted Excel spreadsheet with the study characteristics, including the year of publication, country, study design, the number of randomized participants in each group, participants’ characteristics at baseline, presenting the population of the included studies, age range, and primary diagnosis, EN administration methods, and outcomes of interest. Any disagreement was resolved by a fourth reviewer. Authors of original studies were to be contacted for missing data or further clarifications regarding data collection and/or accuracy. Information from multiple reports of the same study was extracted separately, and we then collated the information from the data collection forms, as recommended by the Cochrane handbook for systematic reviews of interventions [16]. 

### 2.5. Risk of Bias Assessment

The quality of the eligible studies was evaluated using the Revised Cochrane risk-of-bias tool for randomized trials (RoB 2.0) by three independent reviewers. Any discrepancy was resolved by discussion. The overall quality of a study was graded as “low” if all domains were judged to be at low risk. The term “some concerns” was used if at least one domain was considered to have some concerns, but none of the domains were graded with a high risk of bias. A study was graded as “high risk” if at least one domain was judged to be at high risk or more than three domains were judged to have some concerns. The visualization of the risk of bias assessments was performed using a web app entitled “robvis” [17].

### 2.6. Data Synthesis

We applied the vote-counting method [18] according to the direction of effect [19,20] for the outcomes where a meta-analytic model was not feasible to implement. An effect-direction plot was created to synthesize the effect measures and provide a better interpretation of the data [20].

## 3. Results

### 3.1. Selection of Trials

**Figure 1:**
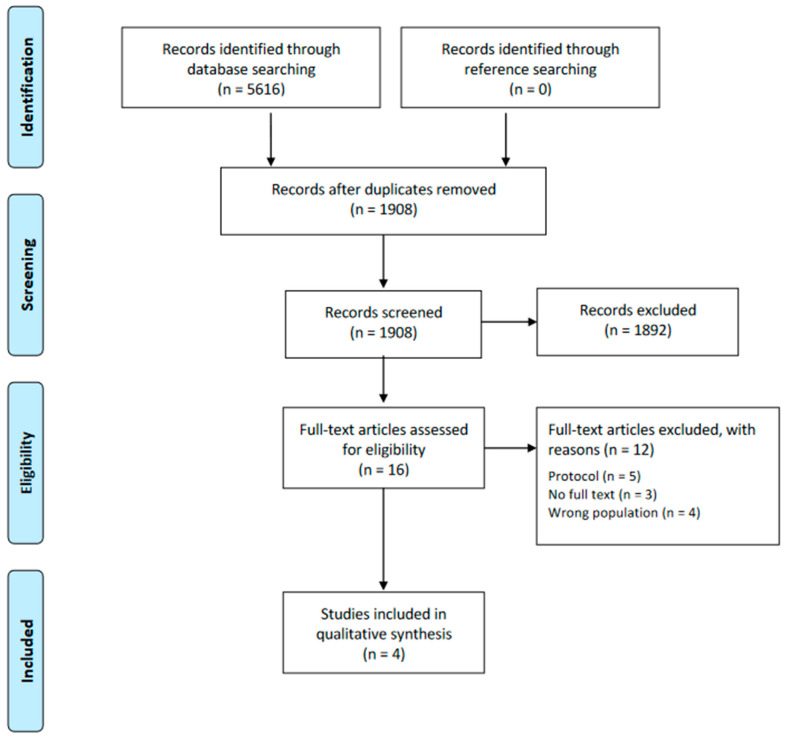
Flow diagram of the eligibility process.

The initial search yielded 5616 records, of which 3708 were removed as duplicates. Of the total 1908 articles, after the title and abstract and the full-text review, four met our inclusion criteria (Figure 1). A comprehensive list of the excluded studies with their corresponding justification can be found in Supplementary Materials (Table S3).

### 3.2. Trial Characteristics

The characteristics of the included studies are presented in Table 1. Of the four included trials, two [21,22] originated from the USA, one from Australia [23], and one from Iran [24]. Half of the studies [22,24] had pre-registered their protocol in a clinical trials registry. One study [22] was unblinded, while the remaining three studies [21,23,24] have not reported their status. All studies were randomized controlled trials of parallel design. One study [22] was a multicenter trial conducted in seven academic PICUs. Study durations ranged from six months [23] to three years [22].

The sample size of the included trials ranged from 25 [21] to 158 [22] children, with a total number of 288 participants, of whom 106 were females. The age of the participants fluctuated from 0 [23] to 17 [24] years old. All patients were admitted to the PICU. Two studies [21,22] reported the primary diagnostic categories of their participants. The majority of patients were categorized as having a respiratory condition, followed by a cardiac disease and infection.

Regarding the administration of EN (bolus or intermittent), two studies used a nasogastric feeding tube [21,22], one study used either the nasogastric or orophagial-gastric feeding route [24], and another study provided information regarding the use of a polyvinyl nasogastric or orogastric gastric feeding tube of a size appropriate for the child’s age [23].

### 3.3. Primary Outcomes

#### 3.3.1. Time Taken to Reach Total Target Caloric Intake

Three out of four [21,22,24] studies evaluated the efficacy of continuous or bolus gastric feeding in achieving total target caloric intake. The study performed by Fayazi et al. [24] reported the time needed to reach the goal caloric intake. There was a difference between the two groups, with patients assigned to the continuous feeding arm reaching the total energy intake in 3.17 ± 1.56 days compared to the bolus feeding group, which required longer (4.35 ± 0.98). In the study by Brown et al. in 2019 [21], the proportion of prescribed energy delivered at 24 and 48 h was approximately 66% and 70%, respectively, in the bolus feeding group, significantly higher than in the continuous feeding group (<40% and <60% for 24 h and 48 h respectively). The latter study by Brown et al. [22] assessed both the time and percentage of energy goals achieved. They revealed a difference in the percentage of energy goals achieved by the patients in the two groups, with the bolus group having a greater percentage of energy goals delivered (78.2%, range: 2.5–207.8%) compared to the continuous group (58.9%, range: 10.9–101.0%). Furthermore, they found that participants in the bolus group had a median time of 18 h to achieve energy requirements compared to 20 h of the continuous group. 

#### 3.3.2. Length of PICU Stay

Half of the studies [21,24] assessed the duration of patients’ hospitalization in the PICU. No difference was found between the two groups regarding the needed days to discharge from the PICU.

#### 3.3.3. Adverse Events

There was a great heterogeneity regarding adverse events reporting in the included studies. A detailed report of the adverse events is presented in Table 2. Emesis was the adverse event that was reported by all trials. Briefly, half [21,22] of the included studies assessed the frequency of emesis as a reason for feeding interruption.

### 3.4. Secondary Outcomes

The majority of our pre-defined secondary endpoints were not reported by any of the included studies. According to our protocol, we aimed to assess anthropometric measures such as children’s head circumference, BMI for age percentile, and body weight. As far as the biochemical indices were concerned, we aimed to evaluate glucose, c-reactive protein (CRP), albumin, and lactate. Finally, the included studies failed to mention the outcomes of newly acquired infections or PICU mortality.

#### 3.4.1. Time Taken to Reach Total Target Protein Intake

None of the included articles evaluated the time needed to reach total protein requirements.

#### 3.4.2. Time of Weaning from Mechanical Respiratory Support

Only one study [21] reported the duration of mechanical ventilation in the two groups. In this study, the bolus group received MC for 5.3 ± 3.4 days, whereas the continuous group for 6.3 ± 8.8 days.

#### 3.4.3. Gastric Residual Volume (GRV)

None of the included studies assessed patients’ GRV following the administration of EN. However, the study conducted by Horn et al. [23,25] measured the distribution of the proportion of patients’ GRV values higher than 5 mL/kg for both study arms.

### 3.5. Risk of Bias Assessment

**Figure 2:**
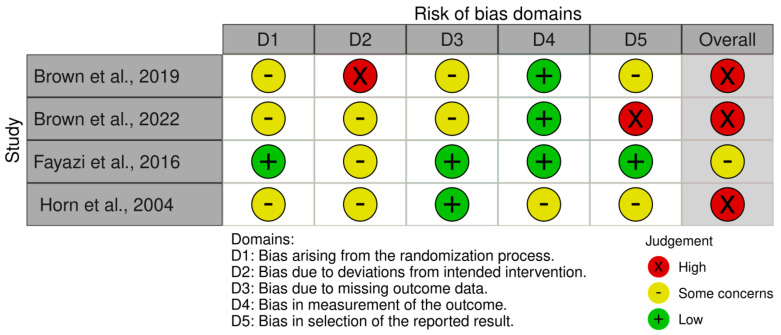
Risk of bias assessment of the included studies [21,22,23,24].

Three of the four included studies [21,22,23] were judged as “high risk” of bias, while the one by Fayazi et al. [24] was deemed as “some concerns”. Figure 2 presents the summary risk of bias for the primary outcomes. The three studies that judged “high risk” of bias [21,22,23], were downgraded in the first and fifth domains and with reference to the second domain, those three studies were also downgraded due to the absence [21,23] or deviations [22] from a prespecified protocol.

### 3.6. Summary of SWiM 

The details of the SWiM summary of the included studies can be found in Table 3. In the two studies, there is no difference between bolus and continuous EN with regard to the length of the PICU stay. As far as the time needed to reach the total energy feeds is concerned, the results are conflicting. It should be mentioned that the study performed by Brown et al. [22] had a larger sample size than the one conducted by Fayazi et al. [24]. According to one study [21], the time to weaning from mechanical ventilation is not affected by the provision method of EN. 

## 4. Discussion

To the best of our knowledge, this systematic review is the first to evaluate the effect of continuous versus bolus-gastric feeding in critically-ill children. Our findings highlight the limited available data, the reporting divergence of the included outcomes, and the high risk of bias in the published studies.

The results of the included records revealed that the length of stay (LOS) in the PICU did not differ between the bolus and the continuous feeding methods [21,24]. As far as the time needed to reach total energy caloric intake is concerned, the results of the available evidence are conflicting due to the differences in methodology, small sample size, and outcome evaluation [22,24]. Results from a cohort study conducted by Martinez and their colleagues, which included 1375 patients in PICU, revealed that 66% of children assigned to the bolus group and 77.4% assigned to the continuous group managed to achieve 60% of the energy requirements within seven days [26]. Interestingly, a number of studies have also evaluated the continuous versus the bolus feeding in adult patients with opposing results [27]. A recently published RCT in critically ill patients found that patients receiving continuous EN had higher achievement rates of the goal nutritional requirement than the intermittent group [28]. However, there are studies reporting the opposite effect, making the results inconsistent for the adult critically ill population [29,30]. 

With regard to the evaluation of GRV, none of the included studies assessed the effect of the different feeding methods on patients’ GRV. It should also be noted that some studies used GRV to evaluate the frequency of feeding intolerance. The use of GRV measurement as a method for evaluating feeding tolerance has been frequently questioned due to insufficient evidence [31]. It has been previously highlighted that frequent assessment of GRV values increases the risk of blocking the feeding tubes and results in decreased nutritional intake; it is hard to interpret and requires additional workload by the staff [32]. A recent survey conducted in 95 PICUs in Great Britain evaluated current practices in GRV measurements and management [33]. It was reported that 42 PICUs were provided written guidance for performing GRV measurements. Thirty-nine units reported measuring GRV at regular intervals, whereas only four reported not conducting GRV measurements. Another study also evaluating GRV practices in the United Kingdom found that 21 out of the 24 PICUS taking part in the survey were using GRV values as the main indicator of EN withdrawal [34]. Notwithstanding, the clinical practice guidelines published by the Society of Critical Care Medicine (SCCM) and the American Society for Parenteral and Enteral Nutrition (ASPEN) do not recommend the use of GRV in ICU patients administered EN [35].

In addition to our main findings, this systematic review demonstrated that children receiving bolus EN have a shorter time of weaning from mechanical ventilation. However, this result has been evaluated and supported only by one study [21] and thus should be interpreted with caution. 

It is of pivotal importance to provide nutrition support to critically ill patients since patients admitted to ICUs and requiring mechanical ventilation support are at higher risk of malnutrition [36], which increases the LOS in the ICUs [37]. Specifically, late initiation of EN is associated with prolonged hospitalization days, LOS in the PICU, and days in mechanical ventilation (DMV) [38]. Furthermore, nutrition support is not only related to the amelioration of clinical-related outcomes such as LOS and DMV but also to the preservation of the patient’s muscle mass by minimizing protein catabolism [39].

The current literature presents high heterogeneity, not allowing for the drawing of safe and robust conclusions. The available evidence assesses outcomes using different measurement methods, for example, the time needed to reach total energy feeds or the proportion of patients reaching the prescribed energy requirements. Furthermore, there is a paucity of evidence concerning the impact of the two main feeding methods on nutritional, anthropometric, and growth outcomes, as well as hard endpoints, such as PICU and within-hospital mortality. Both methods have advantages and disadvantages that should be taken into consideration before decision-making. Namely, bolus feeding is preferred over continuous feeding since it follows a more physiological eating pattern, is cheaper since a pump is not required, and can deliver larger volumes in a shorter time [40]. On the other hand, continuous feeding is preferable and better tolerated when children demonstrate any signs of feeding intolerance or are at high risk of aspiration [41].

The diversity of the reported adverse events among the studies did not allow us to synthesize the side effects of the two feeding methods. Findings from previous studies have revealed that the duration of tube feeding is not related to the reported adverse effects. Furthermore, apart from vomiting, all other adverse events, including nausea, retching, and skin irritation, were not correlated with children’s age [42]. However, the included studies failed to mention long-term adverse events such as nutritional status and growth faltering.

For this systematic review, we performed a search in a clinical trial registry (clinicaltrials.gov, 28 December 2022), and to complement this strategy, we also hand-searched the literature to facilitate and retrieve ongoing trials on this topic. Currently, there are two ongoing protocols involving only pediatric patients admitted in the PICU (NCT02973347 and [43]), categorized as unknown status and ongoing trial, respectively. Additionally, a larger number of registered, active protocols have been identified in adult patients for the comparison of continuous versus bolus-enteral feeding on clinically relevant outcomes. As it has transpired, this research area is still under investigation, and the pending results will provide more data in the near future regarding this topic. We assume that the greater number of active protocols on adult patients is due to the convenience of recruitment and consent procedures for conducting clinical trials.

The strengths of our study are that we used a systematic way to retrieve and appraise the available records by searching in three databases, including gray literature, and using a validated assessment tool, respectively, following the most recent methodological guidelines proposed by the Cochrane handbook. Another strength of our research was identifying evidence gaps and providing recommendations for future well-designed studies in this field. We are willing to update our systematic review when new data is published in the literature.

We should also acknowledge the limitations of this study. The few included studies with great reporting variability of their outcomes, induced heterogeneity, and difficulty in pooling the data together. Moreover, currently, there are only published studies with some concerns or a high risk of biased judgment, reducing confidence in their results. Furthermore, another limitation of our study is that we had a language restriction by including only articles written in the English language. However, a recent systematic review revealed that restricting the search strategy of a systematic review only to articles published in English has little impact on the pooled estimate and conclusion of the study [44].

## 5. Conclusions

Our study shed light on the limited evidence of whether continuous enteral feeding is superior to bolus one in critically relevant outcomes. Future high-quality randomized trials with better methodology and larger sample sizes that examine nutritional, anthropometric, biochemical, and hard outcomes in a homogeneous way, enabling the pooling of the data, are welcomed to guide this decision. Hence, nutrition support teams should take into consideration either feeding prescriptions according to patients’ values and requirements. 

## Figures and Tables

**Table 1:** Characteristics of the included studies.

First Author, Year, Country	Protocol	Masking	Population	Participants (N)	Primary Diagnosis	Age Range	Intervention	Intervention Feeding Route	Comparator	Comparator Feeding Route
Brown, 2022 [22], USA	NCT02566070	Unblinded	Mechanically ventilated PICU patients	158	Cardiovascular, hematologic, infections, injury/poisoning/adverse events, neurologic, oncologic, respiratory		BGF	Nasogastric feeding	Continuous feeding	CGF
Brown, 2019 [21], USA	N/A	N/R	Intubated critically ill PICU patients	25	Respiratory, cardiac, post-operative	1.0–80.3 months	BGF	Nasogastric feeding	Continuous feeding	CGF
Fayazi, 2016 [24], Iran	IRCT201109287655N1	N/R	Pediatric patients hospitalized in the ICU	60	N/A	5–17 years old	Intermittent nutrition method by using NGT or OGT	Nasogastric or Orophagial-gastric feeding	Continuous feeding	Nasogastric or Orophagial-gastric feeding, feeding pump
Horn, 2003 [23], Australia	N/A	N/R	Paediatric patients admitted to the PICU	45	N/A	0.0–153.0 months	Intermittent gastric feeds	Polyvinyl gastric tube of an appropriate size	Continuous feeding	Polyvinyl gastric tube of an appropriate size, feeding pump

BGF: Bolus Gastric Feeding; CGF: Continuous Gastric Feeding; ICU: Intensive Care Unit; IRCT: Iranian Registry of Clinical Trials; N/A: Not Available; NCT: National Clinical Trial; NGT: Nasogastric Tube; N/R: Not Reported; OGT: Orophagial Gastro Tube; PICU: Pediatric Intensive Care Unit.

**Table 2:** Reported adverse events for each arm of the included studies *.

Adverse Events	Horn et al., 2003 [23] Horn et al., 2004 [25]		Fayazi et al., 2016 [24]		Brown et al., 2019 [21]		Brown et al., 2022 [22]	
Number of participants	IF 23	CF 22	IF 30	CF 30	BF 11	CF 14	BF 72	CF 75
Emesis	5 (22.0%)	4 (18.0%)	5 (16.7%)	5 (16.7%)	2 (18.2%)	1 (7.1%)	6 (8.3%)	14 (18.7%)
Diarrhea	9 (39.0%)	6 (27.0%)	14 (46.7%)	18 (60.0%)				
Incidence of food intolerance			18 (60.0%)	9 (30.0%)				
Procedures					1 (9.1%)	1 (7.1%)	2 (2.8%)	6 (8.0%)
Worsening clinical status							4 (5.6%)	2 (2.7%)
Elevated GRV					1 (9.1%)	1 (7.1%)	4 (5.6%)	3 (4.0%)
Consecutive measures of elevated GRV					0 (0.0%)	2 (14.3%)	16 (22.2%)	23 (30.7%)
Elevated GRV and abdominal girth					0 (0.0%)	1 (7.1%)		
Proportion of GRV values above 5 mL/kg	6 (26.0%)	6 (27.0%)						
Other					1 (9.1%)	2 (14.3%)	6 (8.3%)	2 (2.7%)

* Values are presented in frequency (percentage). BF: Bolus Feeding; CF: Continuous Feeding, GRV: Gastric Residual Volume; IF: Intermittent Feeding.

**Table 3:** Effect-direction plot of the outcomes of our interest.

Study	Study Design	Time Taken to Reach Total Target Caloric Intake	Length of PICU	Time to Weaning from MV
Horn et al., 2003 [23]	RCT			
Fayazi et al., 2016 [24]	RCT	▲ ^a^	◀▶	
Brown et al., 2019 [21]	RCT			◀▶
Brown et al., 2022 [22]	RCT	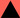 ^b^	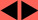	

^a^ Continuous feeding was assigned as the intervention group; ^b^ Bolus feeding was assigned as the intervention group. MV: Mechanical Ventilation; PICU: Pediatric Intensive Care Unit; RCT: Randomized Controlled Trial. Effect direction: upward arrow ▲—positive health impact; small sideways arrows ◀▶—no change/mixed effects/conflicting results; middle sideways arrows 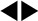
—no change/mixed effects/conflicting results. Sample size: final sample size (individuals) in the intervention group: medium arrow 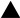 50–300; small arrow ▲ < 50. Study quality (according to RoB 2.0 assessment) denoted by row color: green—low risk of bias; yellow—some concerns; red—high risk of bias.

## Data Availability

Not applicable.

## Supplementary Materials

The following supporting information can be downloaded at: https://www.mdpi.com/article/10.3390/nu15020288/s1, Table S1: Preferred Reporting Items for Systematic Reviews and Meta-Analyses (PRISMA); Table S2: Search strategy for identifying randomized controlled trials on Pubmed; Table S3: Excluded studies with reasons [43,45,46,47,48,49,50,51,52,53,54].
